# Personal KPIs in IVF Laboratory: Are They Measurable or Distortable? A Case Study Using AI-Based Benchmarking

**DOI:** 10.3390/jcm14196948

**Published:** 2025-10-01

**Authors:** Péter Mauchart, Emese Wágner, Krisztina Gödöny, Kálmán Kovács, Sándor Péntek, Andrea Barabás, József Bódis, Ákos Várnagy

**Affiliations:** 1 National Laboratory on Human Reproduction, University of Pécs, H-7624 Pécs, Hungary; krisztina.godony@cdoki.hu (K.G.); kovacs.kalman@pte.hu (K.K.); pentek.sandor@pte.hu (S.P.); barabas.andrea@pte.hu (A.B.); bodis.jozsef@pte.hu (J.B.); varnagy.akos@pte.hu (Á.V.); 2 Department of Obstetrics and Gynecology, Medical School, University of Pécs, H-7624 Pécs, Hungary; wagner.emese@pte.hu

**Keywords:** IVF benchmarking, machine learning, outcome prediction, quality assessment

## Abstract

Background: Key performance indicators (KPIs) are widely used to evaluate embryologist performance in IVF laboratories, yet they are sensitive to patient demographics, treatment indications, and case allocation. Artificial intelligence (AI) offers opportunities to benchmark personal KPIs against context-aware expectations. This study evaluated whether personal CPR-based KPIs are measurable or distorted when compared with AI-derived predictions. Methods: We retrospectively analyzed 474 ICSI-only cycles performed by a single senior embryologist between 2022 and 2024. A Random Forest trained on 1294 institutional cycles generated AI-predicted clinical pregnancy rates (CPRs). Observed and predicted CPRs were compared across age groups, BMI categories, and physicians using cycle-level paired comparisons and a grouped calibration statistic. Results: Overall CPRs were similar between observed and predicted outcomes (0.31 vs. 0.33, *p* = 0.412). Age-stratified analysis showed significant discrepancy in the >40 group (0.11 vs. 0.18, *p* = 0.003), whereas CPR in the 35–40 group exceeded predictions (0.39 vs. 0.33, *p* = 0.018). BMI groups showed no miscalibration (*p* = 0.458). Physician-level comparisons suggested variability (*p* = 0.021), while grouped calibration was not statistically significant (*p* = 0.073). Conclusions: Personal embryologist KPIs are measurable but influenced by patient and physician factors. AI benchmarking may improve fairness by adjusting for case mix, yet systematic bias can persist in high-risk subgroups. Multi-operator, multi-center validation is needed to confirm generalizability.

## 1. Introduction

The assessment of in vitro fertilization (IVF) laboratory performance relies heavily on the use of key performance indicators (KPIs). Metrics such as fertilization rate, blastocyst formation rate, and clinical pregnancy rate (CPR) are designed to provide objective measures of laboratory quality and consistency [1,2]. In most IVF programs, KPIs are routinely calculated at both institutional and individual physician or embryologist level for internal quality management and training purposes [2,3].

The ESHRE Vienna Consensus provides a standardized KPI framework and explicitly acknowledges the use of personal KPIs, while warning that they should not be used for direct performance comparison without adjusting for patient characteristics and case mix [1]. This aligns with findings demonstrating that patient demographics, BMI, age distribution, and treatment indications can substantially distort KPI interpretation and highlight the need for context-aware evaluation [2].

Machine learning and artificial intelligence (AI) are increasingly used in reproductive medicine to predict embryo viability, pregnancy outcomes, and optimize laboratory workflows [4,5,6,7,8,9]. Random Forest and other ensemble learning algorithms have achieved high accuracy (AUC 0.80–0.90) in predicting clinical pregnancy from clinical cycle parameters [6]. Recent work has also suggested using AI-driven KPI monitoring as an early warning system to detect subtle shifts in laboratory culture conditions and individual embryologist performance before they are visible in traditional KPIs [4,5].

In this study, we analyzed three years of Intracytoplasmic sperm injection (ICSI-only) cycles performed by a single senior embryologist to explore whether personal KPIs are truly measurable or inherently distortable. Using both classical statistics and an AI-based Random Forest benchmark trained on the entire clinic dataset, we compared real CPRs against model-predicted expectations across age, BMI, and physician subgroups, and evaluated the impact of indication distribution. This work aims to provide a context-aware framework for interpreting individual embryologist KPIs and highlight the potential and limitations of AI-supported benchmarking in IVF laboratory quality control.

## 2. Materials and Methods

This study is a retrospective, single-operator analysis including all N = 474 ICSI-only cycles performed by one senior embryologist between January 2022 and December 2024 at the Assisted Reproduction Center, University of Pécs, Hungary. The restriction to ICSI cycles was chosen to ensure procedural uniformity, and the results should therefore be interpreted as applicable only to ICSI procedures. For this proof-of-concept case study, we selected the senior embryologist who performed the largest number of cycles during the study period (474/1294 = 36.6%), ensuring adequate power for subgroup analyses. While the AI model was trained on the complete institutional dataset including all operators, the benchmarking analysis presented here was applied to a single operator as an illustrative case study. Only cycles that progressed to embryo transfer (ET) were included in the clinical pregnancy analysis to ensure that the calculated rates reflected transferable embryos. Clinical pregnancy was defined as the ultrasound-confirmed presence of a gestational sac, recorded as either singleton or multiple in the laboratory management system. Cycles without ET were excluded from pregnancy rate calculations but contributed to laboratory process indicators such as fertilization and blastocyst development rates.

Patient characteristics included maternal age, body mass index (BMI), the total FSH stimulation dose, and early-cycle estradiol concentration were recorded. Laboratory parameters comprised the total number of retrieved oocytes, the number of metaphase II (MII) oocytes, germinal vesicles (GV), the number of fertilized oocytes using ICSI, and the number of blastocysts developed from ICSI embryos. Each cycle was also characterized by the IVF cycle number and the treatment indication, categorized into six standard groups: andrological, endometriosis, tubal factor, PCOS, other female, and idiopathic.

From these data, standard key performance indicators (KPIs) were calculated. Fertilization rate was expressed as the proportion of 2 PN embryos among injected MII oocytes. Blastocyst formation rate was defined as the number of blastocysts divided by normally fertilized oocytes (2 PN). Clinical pregnancy rate (CPR) was calculated by the number of clinical pregnancies per embryo transfer cycle.

To assess the relationship between workload and evident performance variability, a Spearman rank correlation was calculated between the monthly number of embryo transfers and the corresponding clinical pregnancy rate.

For assessing the relationship between indication and clinical pregnancy, a logistic regression model was fitted with clinical pregnancy (1 = clinical pregnancy, 0 = no pregnancy) as the dependent variable and treatment indication as the categorical predictor, using andrological ICSI as the reference category. Odds ratios (OR) with 95% confidence intervals (CI) were calculated for each indication. Only embryo transfer cases were included in this analysis to ensure comparability of outcomes. To evaluate the potential cumulative effect of indication distribution on personal KPIs, a stratified Mantel–Haenszel test was applied comparing the clinical pregnancy rates of the selected operator to the institutional dataset across all indication strata (ET-only cases).

Descriptive analysis of CPR was conducted across clinically relevant subgroups. Age was categorized into three groups: under 35 years, between 35 and 40 years, and over 40 years. BMI classification followed WHO cut-offs as a reference. To maintain statistical power with the available sample, the higher BMI ranges were consolidated into two categories: Normal (<25 kg/m^2^), Overweight I (25–30 kg/m^2^), and Overweight II (>30 kg/m^2^). Cycles were also grouped by the treating physician to evaluate physician–embryologist interactions, with all names anonymized for presentation.

A Random Forest classifier was developed as part of the analysis. The model was trained on the entire clinical ICSI-only dataset of the IVF clinic of the University of Pécs, incorporating all cycles from all operators during the study period (N = 1294). This allowed the algorithm to learn population-level relationships between the patient characteristics and laboratory parameters described above and the probability of achieving a clinical pregnancy. The target variable was clinical pregnancy, coded as 1 for pregnant and 0 for non-pregnant outcomes. Model performance was validated using five-fold cross-validation, producing probabilistic predictions for each cycle.

For the single senior embryologist analyzed, the trained Random Forest was used to generate predicted clinical pregnancy probabilities based on the individual case mix and parameter distribution. This approach allowed a direct comparison between the real, observed clinical pregnancy rates and the AI-derived estimates of what would be expected given the patient demographics and cycle characteristics, as learned from the full clinical dataset. Model performance was moderate (ROC-AUC = 0.75; Accuracy = 0.78; Precision = 0.50; Recall = 0.36), reflecting the inherent heterogeneity and class imbalance in a real-world clinical dataset.

Predictors included nine routinely available clinical and laboratory variables: patient age, BMI, baseline FSH dose, early-cycle estradiol level, treatment indication, and the number of retrieved oocytes, as well as laboratory parameters including fertilization rate and blastocyst development. These factors were selected based on their established relevance to IVF outcomes and their consistent availability in the institutional dataset. Feature importance analysis indicated that early-cycle estradiol, BMI, and FSH dose were the most influential predictors, followed by age and oocyte yield, while fertilization and blastocyst parameters contributed at lower levels (Supplementary Figure S1). Observed versus predicted calibration is illustrated in Supplementary Figure S2.

Statistical comparison between the real CPR and AI-predicted CPR was performed using the Wilcoxon signed-rank test. Each embryo transfer cycle generated paired observations: the real outcome coded as a binary value (1 = clinical pregnancy, 0 = no pregnancy) and the AI-predicted probability as a continuous value between 0 and 1. The Wilcoxon test evaluated whether the paired differences between predicted probabilities and observed outcomes were symmetrically distributed around zero. A significant result indicated a consistent directional bias in AI predictions relative to real outcomes. The test was applied separately for maternal age groups (<35, 35–40, >40), BMI categories (Normal, Overweight I, Overweight II), and individual physicians.

To assess agreement between predicted probabilities and observed outcomes across clinically relevant strata, we computed a grouped Hosmer–Lemeshow-type statistic. For each stratum g (e.g., age or BMI group), with n_g_ cycles, observed pregnancies O_g_, and mean predicted probability p_g_, we calculated the expected pregnancies E_g_ = n_g_ × p_g_ and the chi-square statistic

$$C=\sum_{g=1}^{G}\frac{{\left(Og-Eg\right)}^{2}}{Eg(1-Pg)}\;$$

With degrees of freedom df = G − 2, where G is the number of strata. This provides a conservative, strata-level calibration check complementary to AUC. The stratum-level observed and expected values are reported in Supplementary Table S1.

All statistical comparisons between AI-predicted and observed outcomes were assessed at a significance level of *p* < 0.05 and performed with R statistical software ver. 4.51 [10].

## 3. Results

### 3.1. Distribution of IVF Indications 

**Figure 1:**
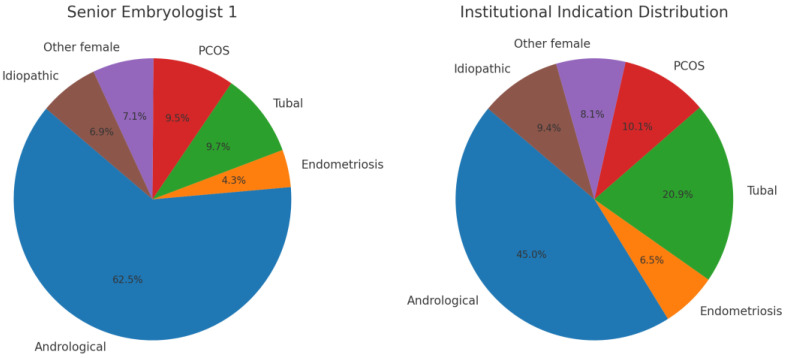
Indication distribution for Senior Embryologist 1 compared with the institutional case mix at the Assisted Reproduction Center, University of Pécs. Pie charts illustrate the relative proportions of treatment indications (Andrological, Endometriosis, Tubal, PCOS, Other female, Idiopathic).

For Senior Embryologist 1, the workload was heavily dominated by andrological ICSI cycles (62.5%), accompanied by a smaller proportion of tubal factor and PCOS cases and a minor idiopathic group (Figure 1). When compared with the institutional indication distribution, the overrepresentation of andrological cases and underrepresentation of tubal factor cycles became evident. This skewed case mix formed the rationale for selecting the dataset of Senior Embryologist 1 as the primary case study for subsequent analyses.

To quantify the potential effect of indication distribution on clinical pregnancy rates, a logistic regression model was fitted using the full institutional dataset, with andrological ICSI as the reference category (Table 1). None of the major indications showed a statistically significant difference in clinical pregnancy odds compared to andrological ICSI. Endometriosis (OR ≈ 1.09, *p* = 0.672) and tubal factor (OR ≈ 1.00, *p* = 0.985) cycles demonstrated comparable outcomes, while idiopathic infertility showed a slight, non-significant trend toward higher odds (OR ≈ 1.13, *p* = 0.540). In contrast, PCOS cases were associated with the lowest odds of clinical pregnancy (OR ≈ 0.38, *p* = 0.121).

The stratified Mantel–Haenszel test adjusting for indication distribution yielded a pooled odds ratio of 1.12 (95% CI 0.83–1.50, *p* = 0.461), suggesting that the overall difference in case mix between the operator and the institutional benchmark did not exert a statistically significant cumulative effect on the clinical pregnancy rate.

### 3.2. Monthly Analysis

**Figure 2:**
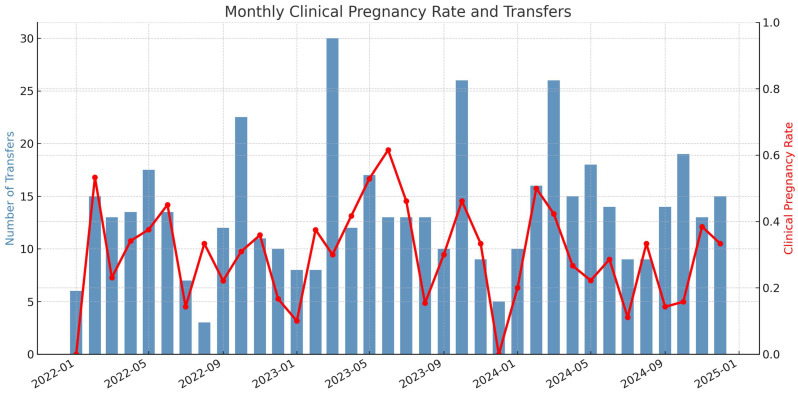
Monthly clinical pregnancy rate (CPR) and number of embryo transfers (ET) performed by the single embryologist between January 2022 and December 2024. Blue bars indicate the number of embryo transfer cycles per month (left Y-axis), while the red line shows the corresponding clinical pregnancy rate (right Y-axis). The figure demonstrates how apparent KPI performance fluctuates markedly in low-volume months (fewer than 10 transfers), with CPRs ranging from near zero to above 0.6. In contrast, months with higher case numbers (>15 ETs) display more stable CPRs around 0.3–0.4. This illustrates the effect of sample size on performance variability and highlights the risk of overinterpreting short-term KPI changes.

Monthly analysis of clinical pregnancy rates (CPRs) and embryo transfer (ET) counts over the three-year dataset revealed marked variability in apparent performance (Figure 2). Months with higher transfer volumes showed relatively stable CPRs, whereas low-volume months displayed pronounced fluctuations, with peaks above 0.6 and troughs near zero. A moderate positive Spearman correlation (ρ ≈ 0.54, *p* < 0.01) between monthly ET count and CPR confirmed that larger case numbers are associated with more consistent outcomes. Cycles with fewer than 10 transfers were especially prone to extreme values, while months with 15 or more transfers typically maintained CPRs in the 0.3–0.4 range. These findings highlight the statistical noise introduced by small sample sizes and illustrate how short-term workload variation can distort the interpretation of personal KPIs.

### 3.3. Age Groups

**Figure 3:**
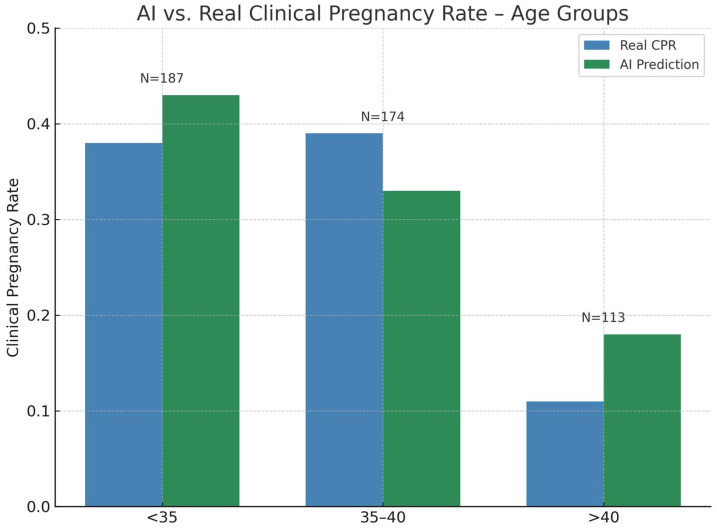
Real versus AI-predicted clinical pregnancy rate (CPR) by maternal age group. Blue bars represent the observed clinical pregnancy rate for each age category, while green bars represent AI-predicted CPRs based on a Random Forest model trained on the full clinical dataset. N denotes the number of embryo transfer cycles in each age group.

Analysis by maternal age revealed systematic but variable differences between real and predicted CPRs (Figure 3, Table 2) In the <35 age group, the AI-predicted CPR (0.43) was slightly higher than the observed value (0.38). The Wilcoxon signed-rank test indicated no significant difference (*p* = 0.346). In the 35–40 category, the real CPR (0.39) exceeded the AI estimate (0.33), with the Wilcoxon test again showing no significant deviation (*p* = 0.860).

In contrast, for patients >40, the AI-predicted CPR (0.18) consistently outperformed the observed real CPR (0.11). The Wilcoxon test identified this difference as statistically significant (*p* < 0.001), highlighting a systematic bias in this high-risk cohort. Grouped calibration indicated a significant miscalibration across age strata (C = 9.01, df = 1, *p* = 0.0027), driven by the >40 subgroup where AI-predicted CPR (0.18) exceeded the observed value (0.11).

### 3.4. BMI Categories

**Figure 4:**
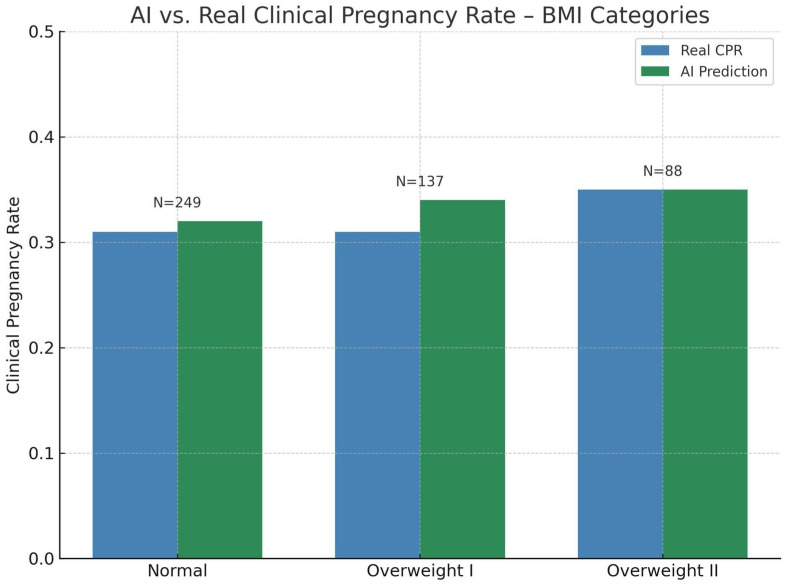
Real versus AI-predicted clinical pregnancy rate (CPR) by BMI category. Blue bars represent observed CPR, green bars show AI-predicted CPRs generated by the Random Forest model. Categories are divided into Normal (<25 kg/m^2^), Overweight I (25–30 kg/m^2^), and Overweight II (>30 kg/m^2^). N denotes the number of embryo transfer cycles per category.

Comparison across BMI categories demonstrated smaller absolute differences (Figure 4, Table 3). In the Normal BMI group, the real CPR (0.31) and AI-predicted CPR (0.32) were numerically close, but the Wilcoxon signed-rank test detected a statistically significant difference (*p* = 0.032), reflecting a consistent directional bias across individual paired values.

For Overweight I patients, the AI-predicted CPR (0.34) was slightly higher than the observed value (0.31), without reaching statistical significance (*p* = 0.072). In the Overweight II category, both real and AI-predicted CPRs were identical (0.35 vs. 0.35; *p* = 0.618). Grouped calibration showed no evidence of miscalibration across BMI strata (C = 0.55, df = 1, *p* = 0.458), indicating that predicted and observed CPRs were well aligned by BMI.

### 3.5. Physicians

**Figure 5:**
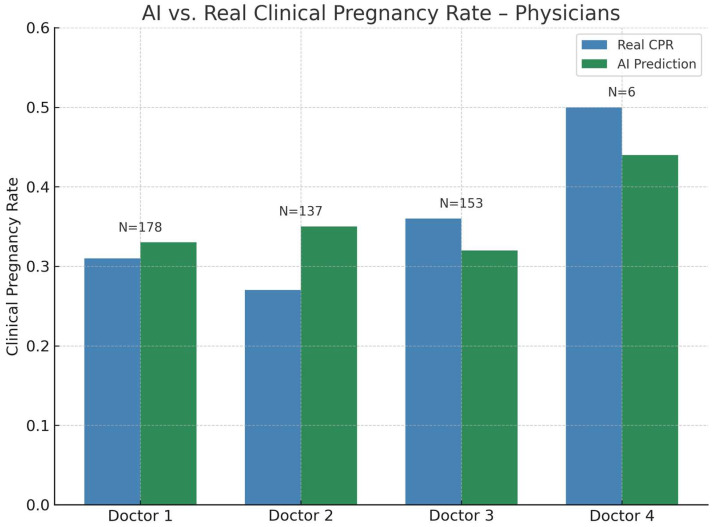
Real versus AI-predicted clinical pregnancy rate (CPR) by physician. Blue bars represent observed CPR, green bars show AI-predicted CPRs for cycles handled in collaboration with each physician. N indicates the number of embryo transfer cycles per physician.

Physician subgroup analysis revealed notable variability (Figure 5, Table 4). For Doctor 1, AI-predicted CPR (0.33) was higher than real CPR (0.31), with a marginal Wilcoxon *p*-value (*p* = 0.056). Doctor 2 showed a larger difference between AI (0.35) and real CPR (0.27), which was identified as statistically significant (*p* = 0.0078).

Doctor 3 displayed the reverse pattern: real CPR (0.36) exceeded AI (0.32), but the difference was not significant (*p* = 0.763). Doctor 4 showed both high real and predicted CPRs (0.50 vs. 0.44) with no significant difference (*p* = 0.844). Grouped calibration by physician suggested no statistically significant miscalibration (C = 5.22, df = 2, *p* = 0.073), consistent with the variability observed in the paired tests, and indicating that physician-level predictions were generally reliable.

## 4. Discussion

To our knowledge, this is the first study to combine AI-based benchmark modeling with paired non-parametric analysis to critically evaluate individual embryologist KPIs while accounting for case mix and treatment indication distribution. Previous studies have proposed KPI frameworks for IVF outcome prediction and quality control [1,2,3], but few have examined their application to personal performance metrics in a single-operator setting [3,4]. Our approach extends this concept by focusing on the dataset of one operator and exploring how AI benchmarks interact with individual case mix.

Indication distribution is a recognized source of bias in personal KPI interpretation. In the present dataset, the operator’s workload was dominated by andrological ICSI cycles (62.5%), whereas the institutional benchmark included a broader indication spectrum with a large proportion of ICSI cycles following failed conventional IVF, representing different prognostic characteristics [2,8]. Contrary to expectation, treatment indication alone did not exert a statistically significant effect on clinical pregnancy odds in the institutional dataset, despite the pronounced skew in case mix between the operator and the benchmark. Logistic regression across all embryo transfer cases found no significant differences between the main indication categories, and a stratified Mantel–Haenszel test adjusting for indication distribution likewise suggested no significant cumulative effect on the clinical pregnancy rate of the operator. These findings indicate only a modest, non-significant influence of case mix in this sample, the observed trends (e.g., lower odds in PCOS, higher in idiopathic infertility) and the clear imbalance in indication distribution reinforce prior reports that case mix can confound KPI assessment and underscore the need to integrate clinical indications into personal KPI evaluation [2,3]. This also supports the use of AI-driven benchmarking to account for context-dependent performance variation [4,5].

Temporal analysis emphasized the role of sample size. Monthly CPRs fluctuated strongly at low case numbers and stabilized at higher volumes. A moderate positive Spearman correlation between monthly embryo transfer counts and CPR confirmed that larger case numbers are associated with more consistent outcomes and that short-term workload variation can distort perceived KPI performance.

One of the most notable findings was the significant difference in the >40 age group, where AI-predicted CPRs consistently exceeded real outcomes. This aligns with previous evidence showing that biological extremes and small sample sizes can lead to KPI volatility and misinterpretation, and highlights the need to consider prediction uncertainty in high-risk subgroups when applying AI-based benchmarks [6]. Advanced maternal age is also well known to be associated with reduced oocyte competence and diminished ovarian reserve [11], elevated rates of embryo aneuploidy that can exceed 70% in women over 35 and rise further in the 40 s [12,13], and impaired endometrial receptivity that contributes to lower implantation and higher miscarriage rates [14]. These biological factors likely explain why the observed CPRs in this group fell short of AI predictions. Conversely, in the 35–40 group, the real CPR exceeded AI predictions, supporting the idea that local workflow or team dynamics can produce performance patterns not captured by population-trained models [9].

The BMI analysis revealed another important point: even when average CPRs appeared similar in the Normal BMI group, the Wilcoxon test identified a consistent directional bias in paired data, underscoring how subtle, systematic deviations can be missed by mean-based metrics. Similar findings have been reported in KPI-score approaches combining laboratory and clinical variables to detect performance shifts at the individual level [3].

Physician subgroup analysis reveals the influence of collaborative patterns on perceived KPI performance. A significant difference for one physician and a marginal effect for another suggest that personal KPIs are shaped as much by case allocation and physician pairing as by laboratory technique. This echoes recent quality control reviews stressing that KPI variability is not solely a function of technical performance but reflects the interplay of clinical and laboratory factors [1,2,9]. Sub-analyses with very small case numbers (e.g., N < 10) demonstrated high variability, highlighting that sufficiently large subgroup sizes are needed to achieve stable and reliable KPI estimates, as small samples can produce substantial apparent variability and bias [15]. Together, these results show that individual embryologist KPIs are measurable but inherently prone to distortion. They reflect technical competence intertwined with patient demographics, treatment indication mix and statistical variance. AI-based models offer a valuable benchmark, yet they can inherit and even amplify existing biases when trained on heterogeneous institutional data and applied to a narrow, unbalanced case set. In particular, since our Random Forest was trained exclusively on institutional data, its predictions may reflect biases specific to the patient population, physician practices, or laboratory protocols of our center [16]. While this provides a realistic benchmark within the local context, external validation on independent multi-center datasets will be required to ensure broader generalizability and to minimize institutional bias [6,7,8]. Beyond discrimination, our grouped calibration analysis provided additional insight into where AI predictions aligned with real outcomes and where they diverged. Across BMI strata, no evidence of miscalibration was found (C = 0.55, df = 1, *p* = 0.458), suggesting that predictions were well calibrated with respect to body mass index. In contrast, age-stratified calibration revealed a significant lack of fit (C = 9.01, df = 1, *p* = 0.0027), driven primarily by the >40 subgroup where AI-predicted CPRs (0.18) consistently exceeded the observed value (0.11). Calibration by physician showed no statistically significant miscalibration (C = 5.22, df = 2, *p* = 0.073), consistent with the paired tests and indicating that physician-level predictions were generally reliable. Taken together, these results show that AI benchmarking works well for most subgroups, but it can still produce systematic bias in high-risk groups. This underlines the importance of checking calibration as well as standard metrics like AUC [17].

AI-based models offer a valuable benchmark, yet they can inherit and even amplify existing biases when trained on heterogeneous institutional data and applied to a narrow, unbalanced case set. In our dataset, the Random Forest model achieved a ROC-AUC of 0.75 and an overall accuracy of 0.78. Precision (0.50) and recall (0.36) were moderate, reflecting the inherent class imbalance and biological variability in real-world IVF outcomes. Taken together, these performance metrics underline both the potential and the challenges of using AI-derived benchmarks for personal KPI evaluation. In addition, continuous refinement of AI models will be required to reduce bias and improve predictive accuracy, particularly in high-risk subgroups such as advanced maternal age patients.

Nevertheless, several limitations should be acknowledged. The relatively small sample size becomes critical when stratified by infertility indications; while the overall dataset was sufficient for the main analyses, subgroup comparisons must be interpreted with caution due to reduced statistical power. Furthermore, the study was restricted to a single embryologist, which inherently limits the generalizability of the findings. This operator was selected because they performed the largest number of cycles, providing adequate power for subgroup analyses. Nevertheless, the methodology should be validated in multi-operator and multi-center datasets to confirm its broader applicability. In addition, degeneration rate after ICSI is another important laboratory KPI reflecting technical micromanipulation performance, and it should be considered in future prospective studies. Other important contributors to IVF outcomes, such as endometrial receptivity [18], embryo morphology [19], and ploidy status, are also well known to affect CPR. In our relatively small, single-operator dataset, including many additional predictors would have oversaturated the model and reduced statistical power; therefore, the analysis was restricted to a focused set of variables.

Taken together, these considerations align with the Vienna Consensus recommendation for adjusting personal KPI evaluation to patient mix and extend it by proposing AI-based benchmarking as a practical, context-aware adjustment tool [1,6,7,8,9].

## 5. Conclusions

Personal KPIs in IVF laboratories offer useful insight into individual performance but are strongly influenced by patient demographics, treatment indication mix, and the effects of small sample sizes. In this single-operator study, AI-based benchmarking demonstrated that apparent KPI differences may reflect case distribution as much as technical skill. Incorporating context-aware, AI-supported benchmarking may improve the fairness and reliability of embryologist KPI evaluation by accounting for patient and cycle characteristics, but further validation in multi-operator and multi-center datasets is needed to confirm its general applicability.

## Figures and Tables

**Table 1:** Logistic regression analysis of treatment indication categories (reference: andrological).

Indication	OR	95% CI Lower	95% CI Upper	*p*-Value
Intercept	0.305	0.258	0.359	0.000
Endometriosis	1.090	0.726	1.637	0.672
Tubal factor	1.003	0.739	1.361	0.985
PCOS	0.377	0.110	1.294	0.121
Other female	0.823	0.587	1.154	0.252
Idiopathic	1.130	0.763	1.674	0.540

OR: odds ratio; 95% CI: 95% confidence interval; *p*-value: significance level of the logistic regression compared to the andrological ICSI reference category.

**Table 2:** Wilcoxon signed-rank test comparing AI-predicted and real clinical pregnancy rates across maternal age groups.

Age Group	N (ET Cycles)	N (Pregnancy)	Real CPR	AI-Predicted CPR	Wilcoxon *p*-Value
<35	187	71	0.38	0.43	0.346
35–40	174	68	0.39	0.33	0.86
>40	113	12	0.11	0.18	**<0.001**

Age group: maternal age category; N (ET cycles): number of embryo transfer cycles; N (Pregnancy): number of clinical pregnancies; Real CPR: observed clinical pregnancy rate; AI-predicted CPR: clinical pregnancy rate predicted by the Random Forest model; Wilcoxon *p*-value: significance of the paired comparison between observed and predicted CPRs. Bold values indicate statistically significant differences (*p* < 0.05).

**Table 3:** Wilcoxon signed-rank test comparing AI-predicted and real clinical pregnancy rates across BMI categories.

BMI Category	N (ET Cycles)	N (Pregnancy)	Real CPR	AI-Predicted CPR	Wilcoxon *p*-Value
Normal	249	77	0.31	0.32	**0.032**
Overweight I	137	43	0.31	0.34	0.072
Overweight II	88	31	0.35	0.35	0.618

BMI category: body mass index group; N (ET cycles): number of embryo transfer cycles; N (Pregnancy): number of clinical pregnancies; Real CPR: observed clinical pregnancy rate; AI-predicted CPR: clinical pregnancy rate predicted by the Random Forest model; Wilcoxon *p*-value: significance of the paired comparison between observed and predicted CPRs. Bold values indicate statistically significant differences (*p* < 0.05).

**Table 4:** Wilcoxon signed-rank test comparing AI-predicted and real clinical pregnancy rates across physician subgroups.

Physician	N (ET Cycles)	N (Pregnancy)	Real CPR	AI-Predicted CPR	Wilcoxon *p*-Value
Doctor 1	178	56	0.31	0.33	0.056
Doctor 2	137	37	0.27	0.35	**0.0078**
Doctor 3	153	55	0.36	0.32	0.763
Doctor 4	6	3	0.5	0.44	0.844

Physician: treating physician; N (ET cycles): number of embryo transfer cycles; N (Pregnancy): number of clinical pregnancies; Real CPR: observed clinical pregnancy rate; AI-predicted CPR: clinical pregnancy rate predicted by the Random Forest model; Wilcoxon *p*-value: significance of the paired comparison between observed and predicted CPRs. Bold values indicate statistically significant differences (*p* < 0.05).

## Data Availability

The data supporting the findings of this study are available from the corresponding author upon reasonable request.

## Supplementary Materials

The following supporting information can be downloaded at: https://www.mdpi.com/article/10.3390/jcm14196948/s1, Figure S1: Feature importance in the Random Forest model trained on the full institutional ICSI-only dataset (excluding the study operator); Figure S2: Observed outcomes plotted against predicted probabilities from the Random Forest model; Table S1: Observed and AI-predicted clinical pregnancy rates (CPR) across subgroups, with observed and expected pregnancy counts used for grouped calibration analysis.
