# Supplementary material for: Personal KPIs in IVF Laboratory: Are They Measurable or Distortable? A Case Study Using AI-Based Benchmarking
Source: J Clin Med. 2025 Oct 1;14(19):6948. doi: 10.3390/jcm14196948 (PMC12525357; doi:10.3390/jcm14196948)

New online supplementary material:

Supplementary Table S1. Observed and AI-predicted clinical pregnancy rates (CPR) across subgroups, with observed and expected pregnancy counts used for grouped calibration analysis.

Stratum	N (ET cycles)	Observed CPR	AI-predicted CPR	Observed pregnancies (O_g)	Expected pregnancies (E_g)
Age <35	187	0.38	0.43	71	80
Age 35–40	174	0.39	0.33	68	57
Age >40	113	0.11	0.18	12	20
BMI Normal	249	0.31	0.32	77	80
BMI Overweight I	137	0.31	0.34	43	47
BMI Overweight II	88	0.35	0.35	31	31
Physician 1	178	0.31	0.33	56	59
Physician 2	137	0.27	0.35	37	48
Physician 3	153	0.36	0.32	55	49
Physician 4	6	0.5	0.44	3	3

Note: Grouped calibration confirmed significant miscalibration by age (driven by the >40 subgroup), no miscalibration across BMI strata, and no significant miscalibration by physician, indicating that physician-level predictions were generally reliable.

Supplementary Figure S1. Feature importance in the Random Forest model trained on the full institutional ICSI-only dataset (excluding the study operator). The model predicts clinical pregnancy using nine clinical and laboratory predictors. The most influential features were early-cycle estradiol level, BMI, and FSH dose, followed by age and total number of retrieved oocytes. Variables related to fertilization and blastocyst development had lower but non-negligible contributions.

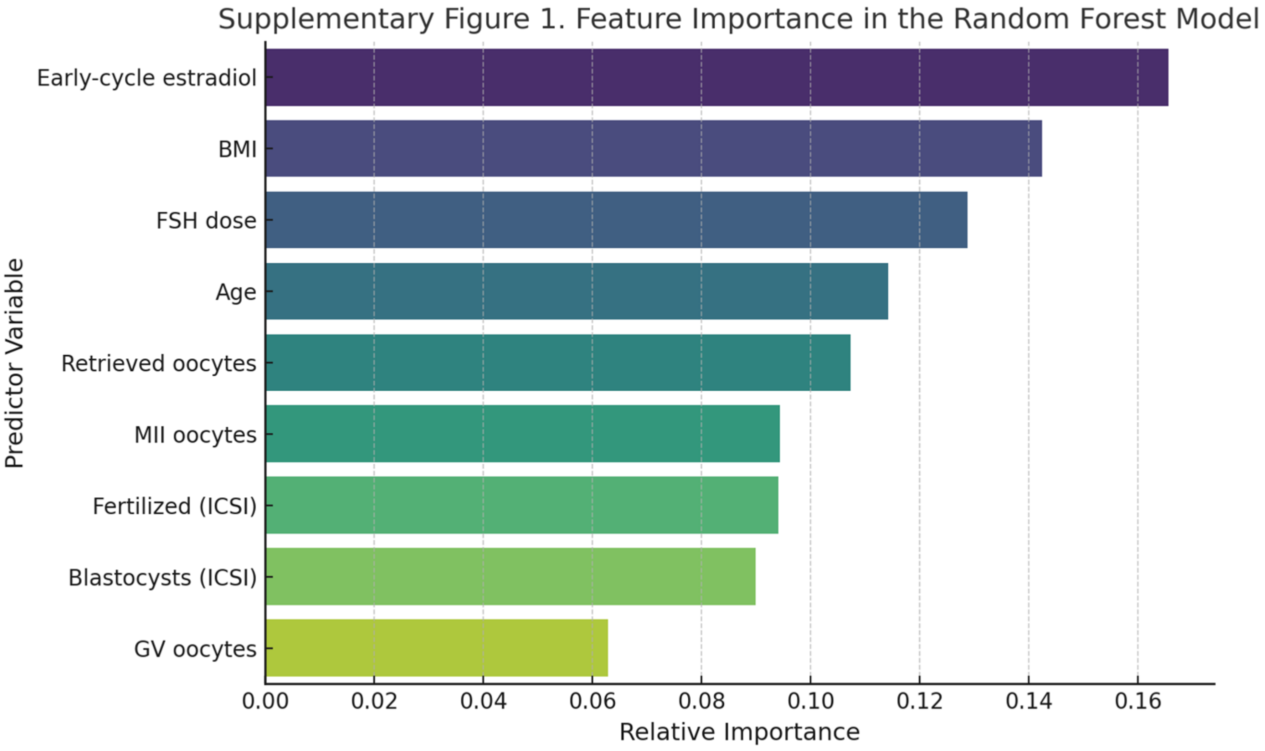

Supplementary Figure S2. Observed outcomes plotted against predicted probabilities from the Random Forest model. Each point represents a single embryo transfer case. The scattered distribution and the visible asymmetry reflect the model's moderate calibration and inherent limitations in classifying rare positive outcomes in real-world IVF datasets.

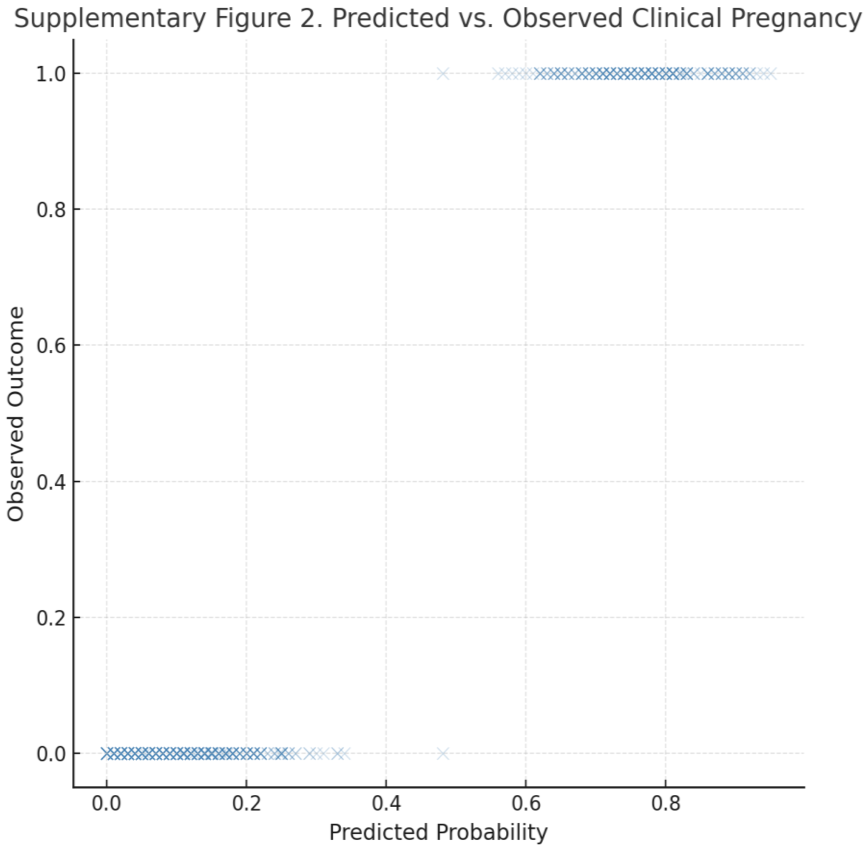

Supplement: Supplementary file 1 [file jcm-14-06948-s001.zip › jcm-3823029-supplementary.pdf]
